# Constitutional Syphilis

**Published:** 1864-12

**Authors:** Jonathan Hutchinson

**Affiliations:** Surgeon to the London Hospital


### SELECTED.

### CONSTITUTIONAL SYPHILIS.

A Clinical Lecture, by JONATHAN HUTCHINSON,

*F. R. C. S., Surgeon to the London Hospital.*

"I sometimes feel almost annoyed at being compelled so very frequently to prescribe iodide of potassium. We go from bed to bed, and to cases apparently of the most different kind, and for almost one in every three I am obliged to dictate the same prescription.* Iodide of potassium in large doses, generally in combination with ammonia, and sometimes with the bichloride of mercury, seems to be the panacea for almost a majority of our cases of chronic disease. Here is a man with convergent squint and double vision; he has come up from Cornwall to be treated, and he looks perfectly healthy. We investigate his case, and pronounce the diagnosis of syphilitic paralysis of his right sixth nerve. A man, a few beds lower down, came in on account of a pain in his heel, which had resisted all treatment for months, and prevented him from either working in the day or sleeping at night. He, too, looked quite healthy; but, on probing his symptoms and history, I gave a syphilitic diagnosis, and, what is more, confirmed it by quickly curing him. A woman was admitted six weeks ago with numerous large ulcers on the legs, and some also on her arms. She had scars of former ulcers about her knees; and the multiplicity of the sores and the worm-eaten character of the edges confirmed the suspicion formed at first glance. She, like the former patient, had had much previous treatment without result, and got well most rapidly under our favorite prescription. There is a boy in Talbot Ward with ascites, and with a liver which reaches below his navel, and with hard periosteal nodes on almost every long bone in his body. His sister was also here not long ago, suffering from nodes; and his mother I have repeatedly had under care during the last fifteen years for various forms of constitutional syphilis. We have also in the same ward two men suffering from chronic enlargement of the testis, which we attribute to the same almost ubiquitous taint. One of them is already nearly well; and the other, I have no doubt, will soon be so.

"If we go down stairs to the woman's ward, we shall find some most interesting cases. There is Mrs. G., the unfortunate wife of a very dissolute sea-captain. She came into the hospital in order to have her sight improved by an artificial pupil, in consequence of adhesions, etc. One of her eyes is shrunk, soft and collapsed; and she has, or rather had, the pupil of the other eye almost wholly closed by lymph. I have made her an artificial pupil, and she sees as much better as we could expect. You will notice that she speaks thickly, uses her limbs awkwardly, and, although not yet middle-aged,

* In mentioning this proportion, I of course allude only to our wards for chronic disease, and do not include those for accidents.

looks as if she were entering on second childhood. Her history is that of a case of subacute syphilitic inflammation of the pia mater. She first came under my observation more than a year ago, at the Moorfields Hospital, for most acute double iritis, and covered with syphilitic rash. The pupils were already covered with lymph, and she was already salivated. We adopted the treatment which seemed best; but, as you see, only with very partial results as regards her eyesight. In her right eye, choroiditis and inflammation of the vitreous body afterward set in; and the eye ultimately became soft, and then shrunken, as it now is. After this, she became exceedingly nervous, could not sleep at nights, and was at length laid up at home with delirium. She was now for some weeks under private care with a form of mania; all her limbs became weak and tremulous; and, when she recovered, those of her left side were weaker than the others. As syphilitic inflammation often attacks the choroid coat of the eye, there is no reason why it should not affect the vascular membrane of the brain; and to suppose that it did so in this instance would well account for all her symptoms.

"In the same ward is a girl aged fifteen, whom we admitted a week ago with large, ragged edged, very deep ulcers on the back of one leg. They are ulcers of a character which, if seen in an adult, you would at once pronounce to be those of tertiary syphilis. And, in confirmation of that view, she has an induration in front of one tibia. The girl is, however, only fifteen; and she has had these ulcers for several years. The disease in her is congenital; and she shows, in order to help us to this opinion, one of the most typical sets of teeth that I have ever seen. You will note that her physiognomy would not have led us to suspect her, for there is nothing very peculiar in it. The bridge of her nose is not flattened; her forehead is not protuberant; nor are there any scars of fissures about her mouth. Her teeth, however, tell the tale, and are so characteristically malformed, that I should venture a positive opinion without other evidence. You will watch the effect of specific treatment upon her ulcers. I will ask you to observe that the ulcers are clearly not due to mere ordinary cachexia, for the girl looks healthy; and should they be well under iodide of potassium in a few weeks, I shall then ask you to remember that they had existed for several years before she came here.

"I have only mentioned about a third of the curious forms of constitutional syphilis at present under our care. You will observe that I omit all primary and secondary forms of disease. Those which we shall at present consider are such only as occur at long periods after the original disease, and come into the category of late tertiary affections. Our knowledge of this latter class has of late years very much improved, and we are now able to recognize many as such which formerly we did not know; and, I am glad to add, we are able to exclude some from suspician which were formerly much suspected.

"The feeling of annoyance to which I adverted, as sometimes arising when one is obliged over and over again to prescribe the same specific, has its origin in a doubt and a fear—a doubt of one's own accuracy of judgment; and, secondly, a fear of the criticisms of others. A sort of fear arises as to whether, after all, the suggestions now and then made, 'Oh, he is riding his hobby--he sees syphilis in everything,' may not have some foundation in truth. Now, this self-mistrust is very natural and very useful in its proper place, but let me warn you not to let it go too far; and, as regards the criticism of others, let me beg of you not to allow them to influence your judgment one iota. There is not the shadow of a doubt that the syphilitic virus is capable of producing effects at extremely remote periods, and after long intervals of apparently good health. There is not the least doubt, further, that this virus is very widely diffused amongst all classes of the community. We must, therefore, expect to encounter its results very frequently, and under very varied circumstances. Our duty in this matter is to find out with accuracy, amongst the great variety of chronic maladies which come before us, which are syphilitic and which are not. Upon our success in diagnosis will depend our success in treatment. There is no room for joking skepticism. It is a simple question of fact. My patient presents a form of disease which we know must have had some cause. We know, further, that the syphilitic taint is a cause quite capable, in some instances, of producing a similar result; and we want to find out by collateral evidence whether that cause is in operation here. And, if it should so turn out that we are obliged, after pains-taking investigation, to believe in the presence and efficiency of that special cause in five out of every twenty patients, it cannot be helped. We want truth; and, if that is the truth, we must take it and act on it. A good means of checking our own conclusions is always at hand. I allude to the results of treatment. In most cases of tertiary syphilis, the consequences of acquired disease, the effects of specific treatment are most prompt and definite. Unfortunately, it is not so in a few, especially in those which concern the nervous system; and it is not so in many which are consequent on inherited taint. In these the efficacy of specific treatment is often but ill marked.

"Before proceeding to relate cases in detail, there are three or four doctrines regarding syphilis which have of late years fought their way to general belief, to which I must ask your special attention.

"The first of these is, that tertiary syphilis may, and often does, last through a person's life. By tertiary syphilis we mean all forms of specific disease occurring subsequently to the primary and exanthematic stages; practically, everything that comes later than two years after the infecting sore. The exanthematic stage usually occurs within two months of the original sore, and is rarely protracted beyond the year. We will, however, to give good margin, say two years. After this the disease appears to have no stages; periods of entire latency, of the most variable lengths, may occur. The symptoms which show themselves are irregular, and subject to repeated relapses after cure by treatment. Between the secondary and tertiary symptoms, an interval of health, often of several years, and it may be of many, supervenes.

"The second cardinal doctrine, as regards tertiary disease, is what I have just adverted to: that it may be *latent*. By latent, I mean that it may be entirely concealed. The patient may appear to be in robust health; may not show the slightest trace of a symptom; may even marry and beget healthy children; and yet the disease may reappear. In a recent lecture I brought forward a case in which the period of latency had been twenty years; and I shall have to mention several others in which it has been nearly as long. The phenomena of latency are even more wonderful in respect to inherited than they are in regard to acquired disease.

"Thirdly, I wish to insist that it is very common for married women to acquire a constitutional taint, without having ever had primary or secondary disease, and, therefore, without either themselves or their husbands having the slightest suspicion of what has happened.* This occurs in women who have borne children to syphilitic husbands, and who have

* According to my own observation, the reverse of this may also occur, and the husband be constitutionally infected without any local lesion or primary disorder.—Z.

imbibed from the fluids of the fœtus the poisonous material. We will call this 'Syphilis by conception.'

"Lastly, we must remark that it is very possible for a patient to have primary syphilis, and never be aware of it. In a woman this may easily be. A small indurated chancre causes very little irritation, and is perhaps never suspected to be of any consequence. It so happens, that the sore most likely to infect gives the least local annoyance; and many an inexperienced man will allow a sore of this kind to go on without treatment, and afterward, in good faith, assure his surgeon that he has 'never had a chancre.' But there are cases even yet more difficult to explain, in which even a practised eye never finds the infecting sore. I have more than once or twice known it to happen, that surgeons or medical students, who came under treatment for secondary forms of disease, and who made not the slightest attempt at secresy, assured me that they had never been aware that they had primary sores.

"The chief lesson to be drawn from these various sources of fallacies in the histories we receive is, that the surgeon must learn, by widely-extended practice, to trust to his own eyes for a diagnosis. The importance of being independent of what our patients may tell us can scarcely be exaggerated in this matter. Not only will it save us from being misled by erroneous statements, but it will in some cases save the necessity for asking painful and annoying questions.

"We will now proceed with some clinical illustrations of our remarks.

"*Latent Syphilis; an Interval of Eight Years without Symptoms, the Patient enjoying Robust Health; Ulcerative Destruction of the Palate, with Psoriasis of the Backs of the Hands.*—Wendon Dawson, aged thirty, a dark-complexioned man, looking much older. Nine years ago he had a sore. He was then in the navy, went into Chatham Hospital; 'took mercury pills, and was salivated freely.' He had a bubo in one groin, which suppurated and remained open for two months. He left the hospital after six weeks, and took no more medicine. He recollects that he had a sore throat, but does not remember any rash.

"On leaving the hospital he went on board ship again; and had good health and remained quite well. Three years later he married. His wife has never conceived, and has remained in perfect health. Very soon after he married he had 'yellow jaundice,' and was very ill for a week or more; he was at home a month. About a year ago his throat began to be sore, and six months after this sore, patches showed themselves on the backs of his hands. He has only been under treatment for these affections for about two months. During the interval since his discharge from the Chatham Hospital, with the exception of the attack of jaundice, he has enjoyed good health, and has been wholly free from symptoms; 'never lost a single day's work.' We questioned him most closely, and cauld not make out that he had had any suspicious symptoms whatever.

"January 13th, 1864. The subject of the above notes was sent to me by Mr. Swales, of Sheerness. He is now cachectic, and speaks in a hoarse whisper. His soft palate is extensively destroyed by ulceration, which is still spreading; his breath has the fetor of diseased bone. The backs of his hands and wrists are covered by patches of psoriasis, with fissures and peeling of epidermis, just like the common psoriasis palmaris. There is not a single patch in either palm. There are two or three similar patches on each cheek. The man states that he has never had any venereal disease since the one described nine years ago, and there is not the least reason to doubt his statement. Let us note also that, although salivated in the first instance, he has never needed any medicine since, except during the last few months. Under about two months' treatment, this patient got quite well as regards his throat; and his psoriasis, although not cured, was much benefited.

"In the next case, we have again phagedæna of the throat, but its chief interest attaches to the fact that the man has entirely lost his hearing. With regard to the throat, I may, however, here ask your attention to the difference between the secondary and tertiary forms of disease as they occur in it. In the secondary stage, the inflammation is always superficial, and always ends in cicatrization, without noticeable loss. Indeed, excepting in the tonsil itself, there is rarely any ulceration. On the velum palati and pharynx it is rather inflammatory swelling than ulceration. All the deeply ulcerative or phagedænic affections of the throat occur years after the primary disease, and are tertiary. Of this, both the cases before us are examples."—(*British Med. Jour.* and *Dublin Med. Press.*)

